# Enhanced Thermoelectric Performance of Cu_2_Se via Nanostructure and Compositional Gradient

**DOI:** 10.3390/nano12040640

**Published:** 2022-02-14

**Authors:** Lin Bo, Fujin Li, Yangbo Hou, Min Zuo, Degang Zhao

**Affiliations:** 1 School of Materials Science and Engineering, University of Jinan, Jinan 250022, China; bling828@163.com (L.B.); happytogetherl@163.com (F.L.); mse_zuom@ujn.edu.cn (M.Z.); 2 Heze Institute of Product Inspection and Testing, Heze 274000, China; garudahou@163.com

**Keywords:** thermoelectric material, nanostructured, interfacial engineering, multi-scale scattering centers

## Abstract

Forming co-alloying solid solutions has long been considered as an effective strategy for improving thermoelectric performance. Herein, the dense Cu_2−*x*_(MnFeNi)*_x_*Se (*x* = 0–0.09) with intrinsically low thermal conductivity was prepared by a melting-ball milling-hot pressing process. The influences of nanostructure and compositional gradient on the microstructure and thermoelectric properties of Cu_2_Se were evaluated. It was found that the thermal conductivity decreased from 1.54 Wm^−1^K^−1^ to 0.64 Wm^−1^K^−1^ at 300 K via the phonon scattering mechanisms caused by atomic disorder and nano defects. The maximum *zT* value for the Cu_1.91_(MnFeNi)_0.09_Se sample was 1.08 at 750 K, which was about 27% higher than that of a pristine sample.

## 1. Introduction

Thermoelectric (TE) technology can convert useless heat into electrical energy, which provides an efficient alternative route for waste heat recycling and refrigeration [1,2,3]. TE material has attracted great attention due to its long working life and high temperature reliability. The conversion efficiency of a thermoelectric device mainly depends on the dimensionless figure of merit (*zT*),

(1) $$zT=\frac{\alpha^{2}\sigma }{\kappa }T$$

where *α*, *σ*, *α*^2^*σ*, *κ* and *T* are Seebeck coefficient, electrical conductivity, power factor, thermal conductivity and absolute temperature, respectively [4,5]. To optimize the *zT* value, band convergence [6,7,8], carrier concentration optimization [9,10], nanostructure [11], as well as interfacial engineering [12,13,14] are designed to simultaneously optimize electrical conductivity and lattice thermal conductivity. Practically, interfacial engineering is an appropriate way to develop high-performance TE materials by inducing nanostructure and manipulating phonon transport properties, which are well aligned with the requirements for good TE materials [15,16,17]. Liu et al., confirmed that the interfacial effect in TE material is represented by the heterogeneous interfaces of matrix/precipitate, which can be strengthened by grain refinement and introducing the dislocations or hierarchical structures [18,19]. So far, the interfacial engineering concept has been extensively used in many TE systems, such as nanocrystalline BiSbTe [16], ZrNiSn [20], SiGe [21], and PbTe [22]. By introducing a nanostructure, the interfacial effect can increase energy filtering effect and hinder phonon transmission, which shows great promise in the manipulation of TE performance. In addition, introducing phonon scattering sources such as grain boundaries (*GBs*) and nanostructure can effectively shorten the phonon relaxation time, resulting in the reduction of *κ_L_* [23,24,25]. Zhang et al., demonstrated that incorporating hetero nano regions can effectively scatter the acoustic phonon and thus induce the low *κ_L_* [10,26,27]. Wang et al., found that the *GBs* between nano-SnTe and Cu_3_SbSe_4_ strengthened the interfacial effect obviously, and the *zT* of 0.71 was obtained at 650 K [12,28]. These results indicated that *GBs* and nanostructure play a significant role in the reduction of *κ_L_* without degrading the electronic transport properties.

Recently, liquid-like thermoelectric materials have been extensively studied due to their great electric transport performance. Compared with the carriers of ordinary thermoelectric materials, the ions of liquid-like materials generally suffer little resistance during migration. This has been well documented in many examples such as Cu_2_Se, Cu_2_S, and Ag_9_GaSe_6_ [29,30]. Chen et al., demonstrated that the TE performance of liquid-like material can be raised by importing various atomic rattling behaviors [31,32], liquid-like ionic migrations [33], or hierarchical structures [34]. Yang et al., found that the phonon scattering of Cu_2_Se was strongly eliminated by nanostructure engineering, and the lattice thermal conductivity was decreased [35]. Peng et al., designed Cu_1.99_A_0.01_Se (A = Fe, Ni, Mn, In, Zn or Sm) alloys and simultaneously decreased the electrical resistivity and the lattice thermal conductivity, resulting in high zT value [36,37]. Most reports focused on introducing a nanostructure into the matrix or single element alloying to improve TE performance of Cu_2_Se material, while there are few works in the literature about the combination of multi-elements co-alloying and interfacial effect.

This work aims to investigate co-alloying Cu_2−*x*_(MnFeNi)*_x_*Se (*x* = 0–0.09) from a thermoelectrically competitive perspective that has not been tried in the past. Multi-scale Cu_2_Se materials were fabricated by a different ball milling (BM) process. The multiple scattering sources induced by interfacial engineering were discussed with the proofs given by transmission electron microscopy (TEM) analyses. It is expected to provide a useful guidance and reference for the industrialization of Cu_2_Se TE materials.

## 2. Materials and Methods

The Cu_2_Se with the nominal chemical compositions was synthesized by vacuum melting. The copper, selenium, manganese, iron and nickel powders (Aladdin, Shanghai, China, ≥99.99%) were mixed uniformly by ball milling and then heated to 1423 K in a sealed graphite crucible, and held for 20 h in a tubular furnace. Different BM time (3, 8 and 16 h) was carried out for the prepared Cu_1.91_MnFeNi_0.09_Se powder, denoted as CS 1, CS 2 and CS 3, respectively. The obtained powders were densified by rapidly hot-pressing sintering (RHP) at 773 K for 30 min under an axial compressive stress of 60 MPa to form disk-shaped samples of *φ*12 × 1.5 mm^2^. Finally, the high-density bulk CS samples were obtained.

The Archimedes method was used to observe the relative density (*d*) of CS samples. Transmission electron microscopy (TEM) analyses were performed on a probe-corrected microscope FEI Talos-F200S (ThermoFisher Scientific, Beijing, China) at 200 KV. The samples for TEM were prepared by focused ion beam (FIB) using the lift-out method. The cross section of specimens used in the fractural analysis was fractured by a bending test. The electrical conductivity (*σ*) and Seebeck coefficient (*α*) of all samples were measured from room temperature to 750 K under a low pressure (~10^2^ Pa) helium atmosphere. All the bulk specimens were discs with the diameter of 12.00 mm and the thickness of 1.00 mm. The Hall coefficient (*R*_H_) was measured using van der Pauw technique under a reversible magnetic field of 1.5 T at room temperature. The carrier concentration (*n*) was calculated using the formula *n* = 1/*eR*_H_, where *e* is the electronic charge. The carrier mobility (*μ*) was calculated via the formula *μ* = *R*_H_*σ*. The thermal conductivity was obtained via *κ* = *λρC_p_*, where *λ*, *ρ* and *C_p_* are the thermal diffusivity, density and the specific heat, respectively. The thermal diffusivity (*λ*) of disk-shaped samples was measured by a laser thermal conductivity meter (LFA-457, Netzsch, Selb, Germany). The specific heat (*C_p_*) of Cu_2_Se that is commonly used was adopted [38].

## 3. Results and Discussion

### 3.1. Effects of Compositional Gradient on the Thermoelectric (TE) Performance of Cu_2_Se

**Figure 1:**
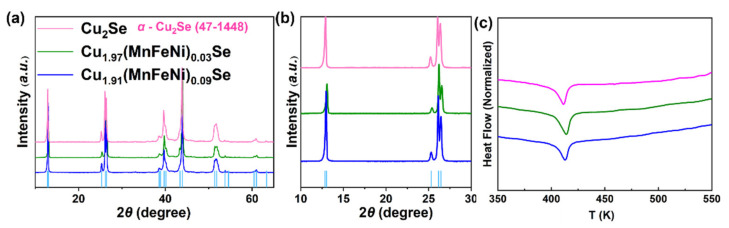
(**a**) X-ray diffraction (XRD) patterns of the bulk Cu_2−*x*_(MnFeNi)*_x_*Se (*x* = 0–0.09) samples. (**b**) Enlarged views of diffraction peaks around 10–30°. (**c**) Differential scanning calorimetry (DSC) curves.

The Cu_2−*x*_(MnFeNi)*_x_*Se (*x* = 0–0.09) materials prepared by vacuum melting and RHP were confirmed by X-ray diffraction (Figure 1a) and indexed based on orthorhombic Cu_2_Se (#47-1448) (a = 13.807, b = 20.393, c = 3.923 Å). The results showed that the *α*-Cu_2_Se phase was the major phase at room temperature, which was consistent with another report [39]. According to the studies by Zhang et al., the *β*-Cu_2_Se phase could appear only when the Cu/Se atomic ratio deviated from the nominal chemical stoichiometry of 2:1 [40]. Previous studies showed that when the temperature approaches the phase transition temperature, the Cu atoms would rearrange and the monoclinic Cu_2_Se transformed into cubic Cu_2_Se, which further resulted in fluctuation in the thermoelectric performance curve [39]. Differential scanning calorimetry (DSC) measurements were carried out for the Cu_2−*x*_(MnFeNi)*_x_*Se samples. As indicated in Figure 1b, an endothermic peak can be found in the DSC curves of the synthesized samples. Therefore, it can be concluded that the samples underwent a transition from *α*-phase to *β*-phase during the heating process.

**Figure 2:**
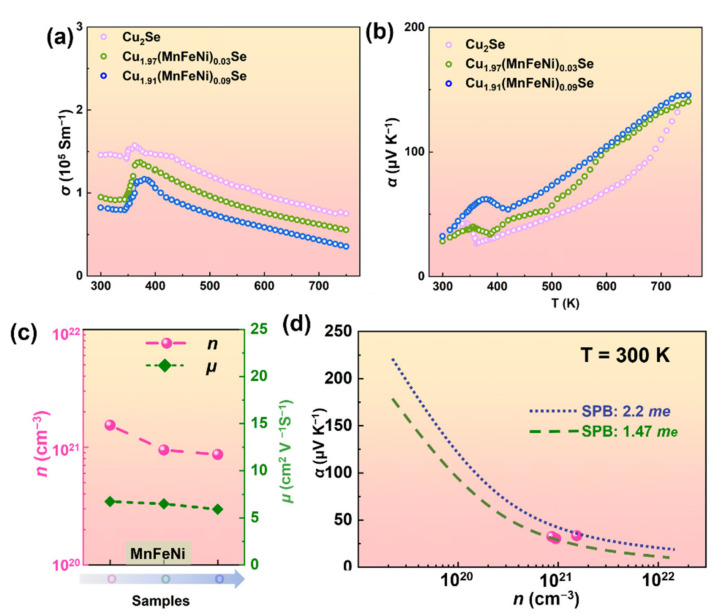
(**a**) Electrical conductivity of Cu_2−*x*_(MnFeNi)*_x_*Se (*x* = 0–0.09) samples. (**b**) Seebeck coefficient. (**c**) Carrier concentration and carrier mobility. (**d**) Pisarenko curve.

The electrical conductivity (*σ*) and Seebeck coefficient (*α*) of Cu_2−*x*_(MnFeNi)*_x_*Se samples are presented in Figure 2a,b, respectively. The electrical performance curves of Cu_2−*x*_(MnFeNi)*_x_*Se samples had an abnormal fluctuation at about 400 K, which was related to the inherent phase transition. The *σ* of all samples decreased with increasing temperature over the high-temperature range, displaying typical doped semiconducting behavior. Pristine Cu_2_Se exhibited a high *σ* of 1.50 × 10^5^ S m^−1^ at 300 K. As can be seen in Figure 2, the substitution of Mn, Fe, Ni (higher valence electrons) on the Cu site resulted in the decrease of carrier concentration (*n*), which decreased from 1.51 × 10^21^ cm^−3^ for Cu_2_Se to 8.68 × 10^20^ cm^−3^ for Cu_1.91_(MnFeNi)_0.09_Se. All Cu_2−*x*_(MnFeNi)*_x_*Se samples kept positive *α* values, which was the characteristic of *p*-type semiconductors. As illustrated in Figure 2b, Cu_2_Se has the lowest value of 146 μV/K at 750 K. To further understand the relationship between *α* and *n*, the Pisarenko curves for Cu_2−*x*_(MnFeNi)*_x_*Se are drawn in Figure 2d. Clearly, the density-of-states effective mass (m*) of Cu_2_Se and Cu_1.91_(MnFeNi)_0.09_Se samples was about 2.20 *m_e_* and 1.47 *m_e_*, respectively. The *α* can be expressed as:

(2) $$\alpha =\frac{\pi^{2}k_{B}^{2}T}{3n}{\left[\frac{\partial \ln\left(\sigma \left(E\right)\right)}{\partial E}\right]}_{E=E_{F}}=\frac{\pi^{2}k_{B}^{2}T}{3n}{\left[\frac{1}{n}\frac{\partial n\left(E\right)}{\partial E}+\frac{1}{\mu }\frac{\partial \mu \left(E\right)}{\partial E}\right]}_{E=E_{F}}$$

(3) $$\alpha =\frac{{8\pi }^{2}k_{B}^{2}}{{3\mathrm{eh}}^{2}}m^{*}T(\frac{\pi }{3n}{)}^{\frac{2}{3}}$$

where *k_B_*, T, μ and *E_F_* are Boltzmann constant, absolute temperature, hole mobility, and Fermi energy, respectively. According to Equation (3), it can be inferred that the decreasing *n* and m* could lead to the constant in *α*.

**Figure 3:**
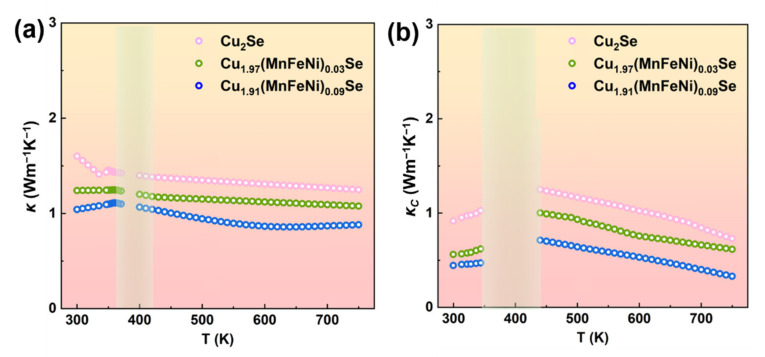
(**a**) Thermal conductivity of Cu_2−*x*_(MnFeNi)*_x_*Se (*x* = 0–0.09) as a function of temperature. (**b**) Carrier contribution to the thermal conductivity of Cu_2_Se.

To verify the characteristics of thermal performance of co-alloying Cu_2_Se, the total thermal conductivity (*κ*) and carrier thermal conductivity (*κ_C_*) are displayed in Figure 3. The *κ_C_* was calculated by *κ_C_* = *LσT*, where *L* is the Lorenz number which can be assessed by the experimental value of *α* according to the SPB model, rather than using a constant value for a degenerate semiconductor, expressed as Equation (4),

(4) $$L={\left(\frac{k_{B}}{e}\right)}^{2}\left\{\frac{\left(\lambda +7/2\right)F_{\lambda +5/2}\left(\eta \right)}{\left(\lambda +3/2\right)F_{\lambda +1/2}\left(\eta \right)}-{\left[\frac{\left(\lambda +5/2\right)F_{\lambda +73/2}\left(\eta \right)}{\left(\lambda +3/2\right)F_{\lambda +1/2}\left(\eta \right)}\right]}^{2}\right\}$$

(5) $$\alpha =\pm \frac{k_{B}}{e}\left[\frac{\left(\lambda +5/2\right)F_{\lambda +5/2}\left(\eta \right)}{\left(\lambda +3/2\right)F_{\lambda +1/2}\left(\eta \right)}\right]-\eta$$

The calculated Lorenz numbers ranged from 1.70 × 10^−8^ V^2^ K^−2^ to 2.40 × 10^−8^ V^2^ K^−2^. As can be seen in Figure 3b, the *κ* of Cu_1.91_(MnFeNi)_0.09_Se was greatly decreased from 1.54 Wm^−1^K^−1^ to 1.02 Wm^−1^K^−1^ at room temperature, which was similar to the results reported by Nunna et al. and can be partly explained by the decreased *σ* and local strain caused by the impurity of the atoms. Compared with the Cu atom, the Mn, Fe, Ni atoms have lower relative atomic mass and smaller atomic radius. Therefore, the stress field and mass field wave scattering were introduced into the Cu_2_Se material when the Cu was substituted by impurity atoms.

### 3.2. Effects of Nanostructure on the TE Performance of Cu_2_Se

**Figure 4:**
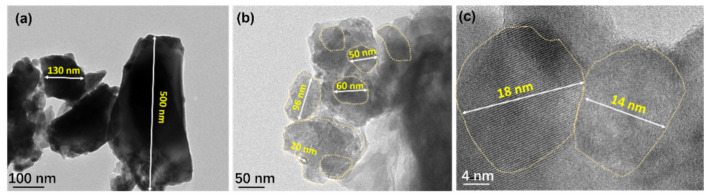
Transmission electron microscopy (TEM) images of CS powder. (**a**) 3 h for CS1. (**b**) 16 h for CS 3. (**c**) Magnified view of CS 3.

To further reduce the thermal conductivity, a series of Cu_2_Se (CS) powders with different particle size were fabricated via ball milling (BM). The corresponding densities of CS powders were 6.56, 6.54 and 6.32 g/cm^3^, respectively. Figure 4a,b display the typical microstructure of CS 1 and CS 3 powders with different grain size. As shown in Figure 4a, most particles of CS 1 were in the range of 100–500 nm. It can be observed in Figure 4b,c, that the agglomerated particles are concentrated within 100 nm (yellow circle), which could possibly provide opportunities for the scattering of medium-range phonons. The particle size of CS 3 could be decreased when the BM time increased, which is beneficial to optimize the thermal performance.

**Figure 5:**
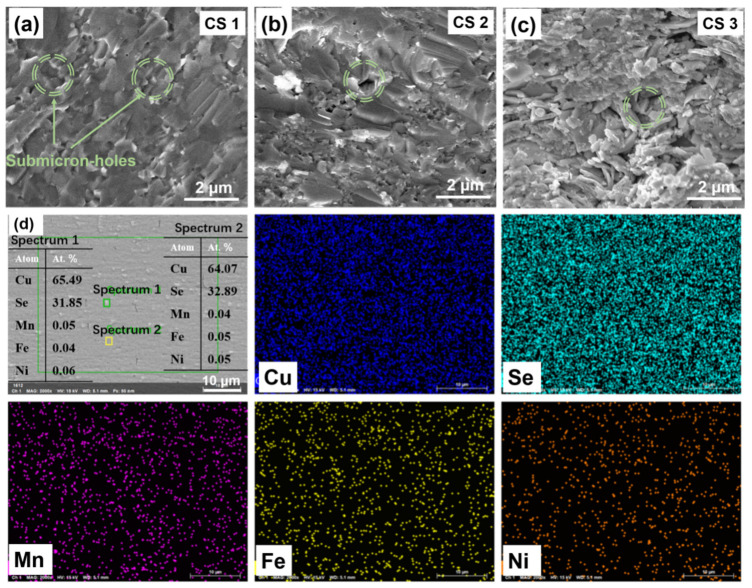
Cross-sectional scanning electron microscopy (SEM) images of CS samples; (**a**) CS 1, (**b**) CS 2 and (**c**) CS 3; (**d**) back-scattered electron image and energy-dispersive spectrometry of the polished surface of CS 3 sample.

For further investigation of the fractured surface of CS (CS 1, CS 2 and CS 3), SEM was carried out. Figure 5a–c present the fractural morphology of the CS sample. The typical microstructure of a layered crystal structure can be observed in the sintered samples, showing a layer-by-layer stacking feature and fixed orientated grains. It can be found in Figure 5 that all samples showed a uniform appearance with distinct boundaries. The grain size of CS 3 was significantly lower than that of CS 1. It also can be seen that there were a few micro-holes and nano precipitates with sizes ranging from hundreds of nanometers to a few microns embedded in the CS sample, as shown in the cyan circle in Figure 5, which can effectively scatter phonons. It can be intuitively understood that massive interfaces were supplied by the abundantly refined grains in CS 3, which could result in significant variation in the transport properties of carriers and phonons. The chemical uniformity and thermal stability of the samples were improved by hot-pressing and repeated annealing processes [41,42]. The actual compositions of Cu, Se, Mn, Fe and Ni in regions 1 and 2 are close to the nominal compositions. It can be seen in Figure 5d that the actual composition of Cu, Se, Mn, Fe and Ni in region 1 and region 2 was close to the nominal composition.

**Figure 6:**
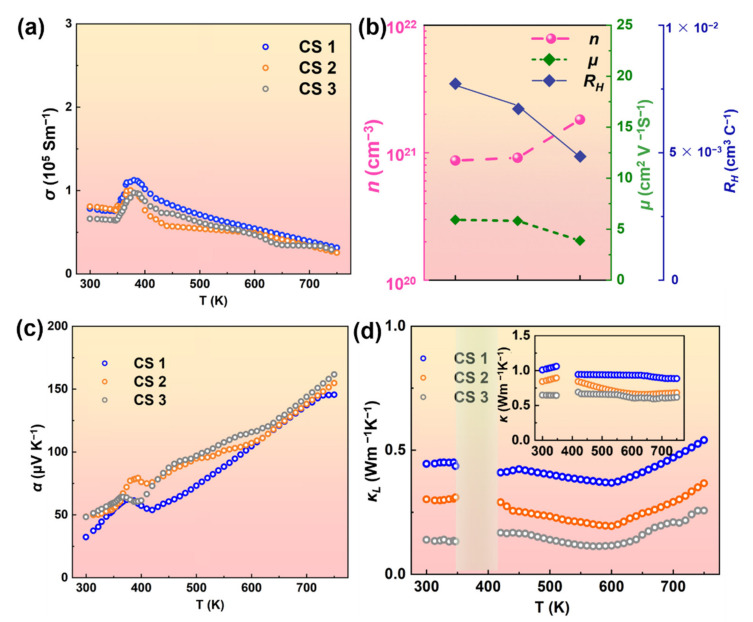
(**a**) Electrical conductivity of CS. (**b**) Carrier concentration, carrier mobility and Hall effect. (**c**) Seebeck coefficient. (**d**) Lattice thermal conductivity of CS, the inset is thermal conductivity.

To assess the influence of the interfacial effect on the carrier transport properties, the variation of carrier concentration (*n*) and electrical conductivity (*σ*) with the reduced grain size are presented, as shown in Figure 6a,b. It can be observed that the *σ* of CS 1 (8.2 × 10^4^ S/m) and CS 3 (7.0 × 10^4^ S/m) remained nearly constant, which should be attributed to the competition between the self-compensation of the vacancy and the reduction of grain size. Similarly, Wang et al., found that the *GBs* could make vacancies more energetically favorable, result in more vacancies, and increase the carrier concentration. To better understand the variation of *σ*, Hall coefficients (*R_H_*) of CS 1–3 were measured. The low mobility could be attributed to the intensive carrier scattering caused by various microstructure defects (point defects and nano precipitates discussed above), which can also be confirmed to some extent by microstructure analysis. The temperature dependence of *α* for the CS samples is shown in Figure 6c. All the samples kept positive *α* values, which was the characteristic of *p*-type semiconductors.

To verify the characteristics of thermal performance for CS, the total thermal conductivity (*κ*) was calculated. The *κ* of CS samples was shown in Figure 6d. The *κ* of CS was greatly decreased from 1.02 Wm^−1^K^−1^ to 0.64 Wm^−1^K^−1^ at 300 K due to the refined grains. A similar result was also found in previous studies about Cu_2_Se samples [37]. Furthermore, it can also be speculated that some inherent properties related to the internal defects/boundary scattering may significantly inhibit the *κ_L_* of CS samples. This variation of *κ_L_* should be related to the defects including nano-holes and aggregated grains with multi-scale, both of which were outcomes of interface engineering. Specifically, the nano-defects could create scattering on low-frequency phonons, while the multi-scale grains induced the plentiful interface at the *GBs*, which can scatter a notable fraction of additional heat-carrying phonons (≥100 nm) with comparable wavelength. It can be speculated that interface engineering was highly practical in decreasing the *κ* due to the severe phonon scattering to enhance the TE performance.

**Figure 7:**
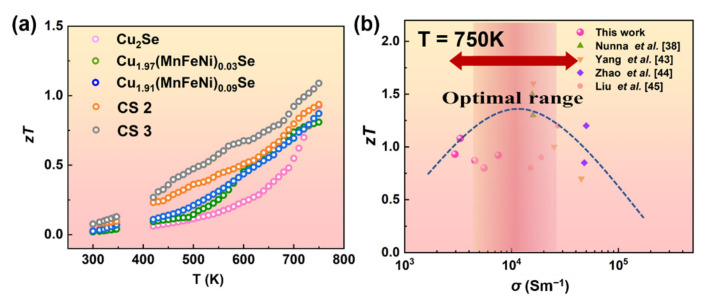
(**a**) *zT* of Cu_2−*x*_(MnFeNi)*_x_*Se (*x* = 0–0.09) as a function of temperature. (**b**) The comparison of *zT* of Cu_2_Se system. The dashed line is calculated on the basis of the single parabolic band (SPB) model.

The *zT* values as a function of temperature are shown in Figure 7. Owing to the highest electrical performance and lowest *κ*, the maximum *zT* of 1.08 at 750 K was obtained for CS 3, which was about 27% higher than that of the un-alloyed sample. Furthermore, to investigate the relationship between *zT* and electrical performance at 750 K, *zT* as a function of electrical conductivity was summarized. The *zT* values of Cu_2−*x*_(MnFeNi)*_x_*Se samples and some experimental data taken from the references [38,43,44,45] were shown in Figure 7b. The experimental data also agreed with the predicted line by the SPB model at 750 K [40]. Experimentally, Cu_2.91_(MnFeNi)_0.09_Se even obtained a high *zT* of 1.08 at 750 K with the extremely low *κ*. It can be found that the interfacial effect can effectively enhance the thermoelectric performance of Cu_2_Se.

## 4. Conclusions

The TE performance of *p*-type Cu_2−*x*_(MnFeNi)*_x_*Se (*x* = 0–0.09) manipulated by compositional gradient and interface engineering was evaluated. It was found that multiple scattering centers of phonons formed via interfacial engineering, including point defects, nano-defects and grain boundaries, which offers an applicable pathway for the reduction of thermal conductivity. The *κ* of CS decreased from 1.02 Wm^−1^K^−1^ to 0.64 Wm^−1^K^−1^ at room temperature without degrading its electrical properties. This work provides an alternative strategy to optimize carrier concentration and suppress thermal conductivity, which is a new method to improve the TE properties for copper-based thermoelectric materials.

## Data Availability

Data are contained within the article.
